# Clinicopathological correlation analysis of (lymph) angiogenesis and corneal graft rejection

**Published:** 2011-06-25

**Authors:** Yongxin Zheng, Haotian Lin, Shiqi Ling

**Affiliations:** 1 State Key Laboratory of Ophthalmology, Zhongshan Ophthalmic Center, Sun Yat-sen University, Guangzhou, China; 2 Department of Ophthalmology, The Third Affiliated Hospital of Sun Yat-sen University, Guangzhou, China

## Abstract

**Purpose:**

To investigate the relationship of (lymph) angiogenesis and survival time of human cornea grafts.

**Methods:**

This was a case series study. A total of 250 patients requiring a second keratoplasty were screened according to our inclusion criteria: 1) only cases with primary non-inflamed cornea diseases were included; and 2) all primary diseased cornea specimens from the first corneal transplantation were confirmed not to have hemangiogenesis or lymphangiogenesis. The included patients were analyzed retrospectively and followed up for the survival time of the first and second grafts. Blood vessel content (BVC) and lymphatic vessel content (LVC) were assessed in the primary diseased cornea; first rejected grafts (including BVC_1_ and LVC_1_) and second rejected grafts (including BVC_2_ and LVC_2_) were assessed by immunohistochemistry. The survival times of the first (STG_1_) and second (STG_2_) rejected corneal grafts were calculated and the relationship between human corneal (lymph) angiogenesis and STG was statistically analyzed.

**Results:**

After screening, only 23 patients (23 eyes) were included. Their primary cornea diseases were non-inflamed, including keratoconus (n=14), leukoma (n=5), and Fuchs endothelial dystrophy (n=4). The mean duration of follow up was 36 months after the second keratoplasty. In all, 55 cornea specimens from different times following penetrating keratoplasty were collected and examined, including 23 primary non-inflamed corneas (without angiogenesis), 23 first rejected corneal grafts (all with hemangiogenesis, but only six cases with blown lymphatic vessels), and nine rejected corneal grafts (including six cases identified with lymphangiogenesis in the first rejection, all with lymphangiogenesis and hemangiogenesis). Based on our statistical analysis, STG_1_ was correlated with LVC_1_ but not with BVC_1_ or (LVC_1_+BVC_1_), while STG_2_ was correlated with (LVC_1_+LVC_2_), LVC_1_, LVC_2_, (LVC_2_+BVC_2_) and (LVC_1_+BVC_1_) but not with BVC_1_ or BVC_2_.

**Conclusions:**

The survival time of human cornea grafts is related to both lymphangiogenesis and hemangiogenesis. Lymphangiogenesis only occurred in some rejected cases, but it seems to be a signal of poor prognosis for the new allograft.

## Introduction

The major cause of corneal allograft failure is considered to be immunological rejection [1-5], which depends on both hemangiogenesis and lymphangiogenesis. These co-occur as two arms of an immune reflex arc and accelerate immune reactions leading to corneal rejection [6-8]. Numerous studies have been conducted on the processes of corneal blood vessel proliferation, whereas reports on corneal lymphangiogenesis are relatively scarce, especially for transplanted human corneas [8,9]. We previously reported that lymphangiogenesis occurred after corneal transplantation in both rats and humans [10]. However, without detailed clinical data, the relationship of angiogenesis and graft survival time cannot be determined, nor can the system (lymphatic system or blood vessels) that plays the most important role in accelerating graft rejections [5,11,12] be determined. In the present research, we began our clinical study by screening out cases of hospitalized patients who had previously received penetrating keratoplasty because of non-inflamed corneal diseases (taken to mean no hemangiogenesis or lymphangiogenesis) [13], but now have experienced corneal graft rejection and require a second keratoplasty. All of the included patients were followed up until the second corneal allograft failed and needed a third transplantation or ophthalmectomy. Each cornea specimen was assessed for blood vessel content (BVC) and lymphatic vessel content (LVC) by immunohistochemistry with antibodies specific for CD31 (vWF) and the lymphatic endothelial markers (lymphatic vessel endothelial hyaluronan receptor [LYVE-1]). The survival time of the graft (STG) was recorded and statistically analyzed together with the pathology results.

## Methods

In total, 250 hospitalized patients, who required a second keratoplasty because of graft failure occurring during January 2005 to December 2008, were screened. Only patients who met the inclusion criteria were included in our study. The protocol and informed consent forms were reviewed and approved by the Institutional Review Board/Ethics Committee of Sun Yat-sen University and a written informed consent form was completed by each study participant. The study was conducted in accordance with the Declaration of Helsinki and the ethical standards of the local ethics committee.

### Inclusion criteria

Primary diseases for the first keratoplasty were non-inflamed cornea diseases. No systemic immune diseases were found and no immunosuppressant was used preoperatively. All of the original diseased cornea specimens (obtained from the first corneal transplantation) from the included patients were confirmed to be without hemangiogenesis and lymphangiogenesis by immunohistochemistry [6,14] and all cornea specimens were identified to be vWF^-^/LYVE-1^-^(Detailed methods are described below). All of the patients received second or third penetrating keratoplasty within one week after identification of signs of graft rejection (The time interval between the first and second, or second and third penetrating keratoplasty could be considered as the survival time of the first or second corneal allograft.). All of the diseased/rejected corneas were available and were collected, and all of the included patients provided signed consent and could be followed up according to our schedule.

### Surgical and clinical follow-up protocol

Penetrating keratoplasty was performed on all included patients according to standard methods [1] and they received the same immunosuppressive agents after each keratoplasty. The  detailed regimen included:

1: 0.5% Tacrolimus Eye Drops, Qid for three months;
2: Compound Tobramycin Eye Drops: 0.3% tobramycin + 0.1% dexamethasone, Qid for one month;
3: Oral Predinisone 1mg/(kg/d), Qid, tapering by 20% every week, use for four weeks.
After the second keratoplasty, patients were followed up every four weeks (one month) until the second corneal allograft failed and they required a third transplantation or ophthalmectomy.

### Immunohistochemistry

After corneas were sent to our Ocular Pathology Laboratory for histopathologic evaluation, they were fixed in 10% neutral formalin for 24 h, embedded in paraffin and sectioned (4 μm sections: 20 sections were from the central part of each cornea and the other 20 slices were from peripheral part). All sections of rejected corneas were rehydrated with graded ethanol-water mixtures, washed with distilled water, and then endogenous peroxidase activity was blocked by incubation with 30 ml/l hydrogen peroxidase for 20 min. Every two neighboring sections were selected and stained with streptavidin-biotin complex. The chosen cornea sections were incubated for 3 h with mouse anti-human LYVE-1 polyclonal antibody (R&D Systems, Minneapolis, MN) and mouse anti-human cluster of differentiation 31 (CD31; R&D Systems). The slices were incubated with biotin-marked rabbit anti-mouse immunoglobulin (Boster, Wuhan, China) as the secondary antibody. Finally, after incubation with streptavidin-biotin horseradish peroxidase (SAB-HRP) complex (Boster) at room temperature for 30 min, the slides were visualized for peroxidase activity with diaminobenzidine and counterstained with hematoxylin.

### Lymphatic vessel content and blood vessel content

Lymphatic vessel content (LVC) and blood vessel content (BVC) of the human corneas were evaluated independently by two observers who had no prior knowledge of the experimental details, and the evaluations were repeated twice. The CD31^(+)^ LYVE-1^(-)^ stained vessels in cornea slices were identified as blood vessels, whereas the CD31^(+)^ LYVE-1^(+)^ vessels were identified as lymphatic vessels. Each cornea sample was sliced into 40 slices (20 slices were from central part and the other 20 slices were from peripheral part of each cornea). The BVC was calculated by summing all blood vessels in the 40 slices and dividing by 40. Similarly, LVC was calculated by summing all lymphatic vessels in the 40 slices and dividing by 40 [6,8,14].

### Statistics

Analysis of the significance of difference was performed using the paired Student’s *t* test for normally distributed data (BVC_1,_ LVC_1_+BVC_1_ and STG_1_) and ANOVA for non-normally distributed data (STG_2,_ BVC_2_, LVC_1_, LVC_2,_ LVC_2_+BVC_2_; with the use of SPSS software [version 17.0; SPSS, Inc., Chicago, IL]). A multivariate model for STG was developed. Values are presented as mean±SD. Differences were accepted as significant when p<0.05.

## Results

### Included patients cohort

Screening of 250 patients with corneal graft rejection identified only 23 patients (23 eyes, 15 male and 8 female; mean age, 50±14 years; range, 18–69) for inclusion in the study. Primary non-inflamed cornea diseases included keratoconus (n=14), leukoma (n=5), and Fuchs endothelial dystrophy (n=4). After the second keratoplasty, patients were followed up every month. The mean duration of follow up was three years (average 36 months, 26–42 months) after the second keratoplasty. In nine cases, the second transplanted corneas were rejected during our follow up. (In these 9 cases of second rejection, 4 patients received a third corneal transplantation, but the other 5 patients had to undergo ophthalmectomy for lack of any better choice.) The other 14 cases of second transplanted corneas are still functioning. In all, 55 cornea specimens from penetrating keratoplasty or ophthalmectomy, including 23 non-vascularized corneas (primary non-inflamed diseases), 23 first rejected corneal grafts, and nine rejected corneal grafts, were obtained for examination in this study. 

### Survival time of grafts (STG)

**Figure 1:**
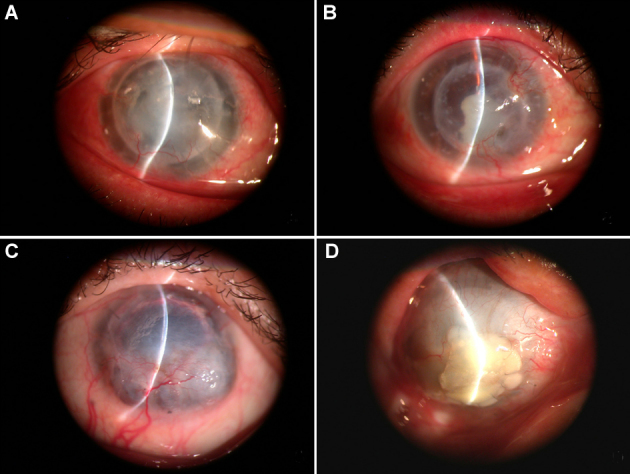
Slit lamp photographs of different rejected corneal grafts. **A**: Transplanted cornea was rejected with slight hemangiogenesis occurring and light corneal edema, the iris and pupil are difficult to discern. **B**: Transplanted cornea was rejected and showed evident hemangiogenesis and slight corneal edema, the iris and pupil were blurry but could be distinguished. **C**: A rejected transplanted cornea is scattered with thick hemangiogenesis; the iris and pupil cannot be seen. **D**: The rejected cornea had a perforation with thick hemangiogenesis and severe corneal edema.

According to our designation, the survival time of the first corneal allograft (STG_1_) was estimated as the time interval between the first and second penetrating keratoplasty (23 eyes, 18.478±7.089 months; range, 3–33 months). In these 23 rejected allografts, 17 cases presented mild hemangiogenesis (Figure 1A), but the other six cases presented severe hemangiogenesis and inflammation (Figure 1B), as determined by slit lamp examination. The survival time of the second corneal graft (STG2) was calculated as the time interval between the second and third penetrating keratoplasty or ophthalmectomy (nine eyes were rejected, 9.000±5.477months; range, 2–17 months; the other 14 cases are still functioning). In these second rejected cases, four patients received a third corneal transplantation (Figure 1C), but the other five patients had to receive ophthalmectomy (Figure 1D) for lack of a better choice.

### Corneal blood and lymphatic vessels in rejected cornea grafts

**Figure 2:**
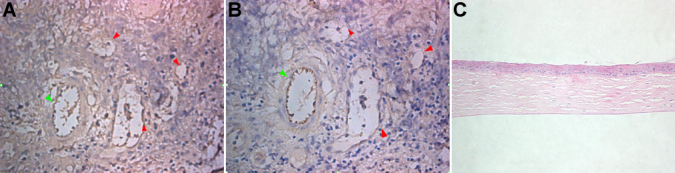
Anti-LYVE-1 and anti-CD31 immunochemistry. **A**: Immunochemistry with CD31 as the first antibody. All vessels show  brown endothelium (red and green arrow). **B**: Immunochemistry with LYVE-1 as the first antibody; two vessels were the counterpart of the two in the left figure, one shows a brown stained lymphatic vessel (green arrow), and the other one was inferred to be a blood vessel (red arrow). **C**: Normal human cornea was absent of blood vessels and lymphatic vessels.

The first antibody for CD31 for immunohistochemistry revealed a positive (brown) staining for CD31 in lymphatic vessels and blood vessels (brown), as shown in Figure 2A. The first antibody for LYVE-1 in a neighboring cornea slice showed positive (brown) stain for lymphatic vessels but blood vessels were negative (no staining) for LYVE-1, as shown in Figure 2B. The brown corneal lymphangiogenesis appeared like vessels or strips, along with blue vessels that were scattered within the positive stroma [6,8,10,14]. From the first corneal transplantation, none of the primary cornea specimens with original diseases showed hemangiogenesis or lymphangiogenesis. From the second corneal transplantation, we found that 17 cases of the first rejected cornea that presented with mild hemangiogenesis by slip lamp examination (Figure 1A), which was confirmed by immunohistochemistry that showed hemangiogenesis occurring and inflammation cells infiltrating. However, the other 6 cases of first rejected corneas (Figure 1B) showed both hemangiogenesis and lymphangiogenesis. All nine cases (Figure 1C,D) of second rejected transplanted cornea were confirmed to show hemangiogenesis and lymphangiogenesis.

### Clinicopathological correlation

**Figure 3:**
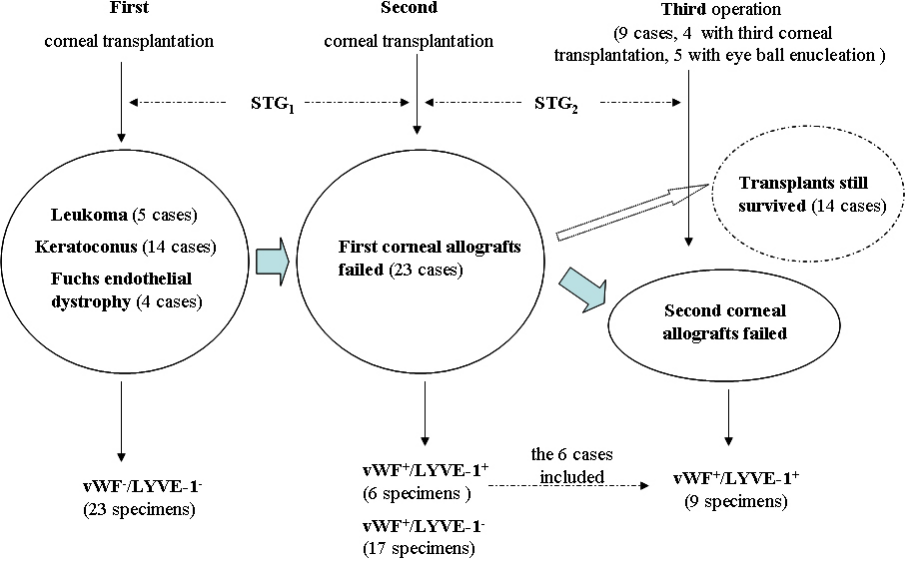
Schematic diagram of survival time of grafts (STG), hemangiogenesis and lymphangiogenesis detected at different times following corneal transplantation.

The relationships of STG, hemangiogenesis, and lymphangiogenesis at different times of corneal transplantation are shown in Figure 3.

The clinical data of STG_1_ and STG_2_ and the pathological results of immunochemistry for LVC and BVC allowed us to perform statistical analysis of the relationship of angiogenesis and graft survival time and to determine which one (lymphatic or blood vessels) may play the most important role in promoting corneal graft rejections. The paired Student *t* test was used to compare the correlation between STG and LVC, BVC or both (Table 1). The STG_1_ showed a significant correlation with LVC_1_ (Correlation=-0.577, p=0.004) but not with BVC_1_ or (LVC_1_+BVC_1_); STG_2_ has a significant correlation with (LVC_1_+LVC_2_), LVC_1_, LVC_2_, (LVC_2_+BVC_2_) and (LVC_1_+BVC_1_) (all with p<0.05, and the correlation coefficients were all negative, with values from largest to smallest.) but not with BVC_1_ or BVC_2_ (p>0.05). Based on our statistical analysis, we could see that the survival time of human cornea grafts (STG) was related both with lymphangiogenesis (LVC) and hemangiogenesis (BVC), but lymphangiogenesis (LVC) may play a more important role.

**Table 1:** Correlation between STG and LVC, BVC or (LVC+BVC).

**Variables**	**Correlation coefficient**	**Significance (p)**
STG_1_ & LVC_1_	−0.577	0.004*
STG_1_ & BVC1	0.058	0.794
STG_1_ & (LVC_1_+BVC_1_)	−0.249	0.251
STG_2_ & LVC_2_	−0.825	0.006*
STG_2_ & BVC_2_	−0.618	0.076
STG_2_ & (LVC_2_+BVC_2_)	−0.821	0.007*
STG_2_ & LVC_1_	−0.885	0.002*
STG_2_ & BVC_1_	−0.125	0.794
STG_2_ & (LVC_1_+BVC_1_)	−0.688	0.041*
STG_2_ & (LVC_1_+LVC_2_)	−0.936	0.000*

*When p<0.05, paired T-Test is significant and the correlation is accepted. ^†^STG=The survival time of graft, LVC=Lymphatic vessels counting, BVC=Blood vessels counting. ^††^1 represents the first corneal allograft, 2 represents the second corneal allograft.

## Discussion

A normal-risk keratoplasty usually has a successful outcome and this is attributed to corneal immune privilege [5]. The lack of both afferent lymphatic and efferent blood vessels in the cornea prevents operation of either arm of an immune reflex arc and is the most important cause of privilege among all factors [11,12,15,16]. For a high-risk keratoplasty, preexisting corneal stromal blood vessels have been identified as a strong risk factor for immune rejection after corneal transplantation, both in the clinical setting and in the well-defined mouse model of corneal transplantation [11,15,17]. Recently, in addition to blood vessels (the efferent arm of an immune reflex arc: access for immune effectors cells to the graft), lymphatic vessels that are microscopically undetectable have been found in association with blood vessels in vascularized high-risk human corneas [8]. These lymphatic vessels enable effective access of donor and host antigen-presenting cells (APCs) and antigenic material to regional lymph nodes, where accelerated sensitization to graft antigens occurs (the afferent arm of an immune reflex arc) [5-8,18]. Yamagami et al. [19] have successfully inhibited allograft rejection of mice after obstruction of corneal lymph drainage by bilateral cervical lymphadenectomy [20,21]. Their later study showed that the survival rate of corneal grafts placed into vascularized recipient beds was as high as 92%^21^, which suggested that lymphangiogenesis plays a more important role in allograft rejection than does corneal hemangiogenesis in animal models.

Research into human corneal lymphangiogenesis has been scarce until now. Claus Cursiefen first reported that lymphatic vessels existed in vascularized human corneas [8]. In his report, lymphatic vessels were found in 10 out of 21 cases of vascularized human corneas caused by different diseases. In our previous study [10], we also reported that lymphangiogenesis occurred in mouse and human transplanted corneas, but the clinical role of lymphangiogenesis in allograft rejection (accelerating or preventing rejection) still awaited identification [5,21]. Many immunology related questions are still unanswered, such as:  whether preexisting corneal stromal blood vessels are an essential condition for the lymphangiogenesis process; whether lymphangiogenesis implies inevitable corneal graft rejection; the nature of the relationship of angiogenesis and graft survival time; and which (lymphatic or blood vessels) may play a more important role in accelerating corneal graft rejections. The present study was designed to answer these questions by clinicopathological analysis of angiogenesis and corneal graft rejection.

According to our inclusion criteria, only cases with primary non-inflamed cornea diseases were included. All of the primary diseased cornea specimens from the first corneal transplantation were confirmed to show no hemangiogenesis or lymphangiogenesis. Therefore, the first corneal transplantation could be considered as a normal-risk keratoplasty and preexisting corneal stromal (lymph) angiogenesis is apparently not an essential condition for allograft rejection. However, for the second corneal transplantation, we found that 17 cases of rejected allografts were confirmed with only hemangiogenesis occurring and with immunochemistry showing infiltration with inflammation cells, while six cases of rejected corneas showed both hemangiogenesis and lymphangiogenesis processes. The positive rate of lymphangiogenesis was 26.1%, which is much lower than the value reported in vascularized human corneas (48%) by Cursiefen et al. [8]. Two reasons may explain this: First, all patients received a second penetrating keratoplasty within one week of identifying the sign of the graft rejection, so the time may be not have been long enough for development of lymphangiogenesis. Second, the primary cornea diseases of all the cases in our study were non-inflammatory, without hemangiogenesis or lymphangiogenesis. Lymphangiogenesis may be induced by or lead to allograft rejection, which should be further confirmed in the future study. Based on the third surgery, all nine cases of second rejected allograft were confirmed with hemangiogenesis and lymphangiogenesis. In these nine cases, six cases had been identified with preexisting corneal stromal hemangiogenesis or lymphangiogenesis from the second cornea transplantation, and the STG_2_ is obviously shorter than the other allograft. Lymphangiogenesis only occurred in some rejected cases, but it seems to be a signal of poor prognosis for the new allograft.

We investigated the relationship of lymphangiogenesis and corneal graft rejections further by statistical analysis of the correlation of STG, LVC, and BVC of different corneal allografts. We found that both hemangiogenesis and lymphangiogenesis were significantly correlated with STG. The second corneal grafts, in particular, when placed into lymphatic-vascularized recipient beds, would fail quickly with additional and more severe lymphangiogenesis and hemangiogenesis. In other words, similar to the implication of lymphangiogenesis for tumor metastasis [13,22-24], new allograft rejection is predictable and inevitable when lymphangiogenesis is found in corneal recipient beds [11,25].

In conclusion, the survival time of a human cornea graft is associated with both lymphangiogenesis and hemangiogenesis, and lymphangiogenesis is a signal of poor prognosis for the new allograft.

## References

[1] Küchle M, Cursiefen C, Nguyen NX, Langenbucher A, Seitz B, Wenkel H, Martus P, Naumann GO (2002). Risk factors for corneal allograft rejection: intermediate results of a prospective normal-risk keratoplasty study.. Graefes Arch Clin Exp Ophthalmol.

[2] Plsková J, Duncan L, Holán V, Filipec M, Kraal G, Forrester JV (2002). The immune response to corneal allograft requires a site-specific draining lymph node.. Transplantation.

[3] Sano Y, Ksander BR, Streilein JW (1995). Fate of orthotopic corneal allografts in eyes that cannot support anterior chamber-associated immune deviation induction.. Invest Ophthalmol Vis Sci.

[4] Streilein JW, Yamada J, Dana MR, Ksander BR (1999). Anterior chamber-associated immune deviation, ocular immune privilege, and orthotopic corneal allografts.. Transplant Proc.

[5] Cursiefen C (2007). Immune privilege and angiogenic privilege of the cornea.. Chem Immunol Allergy.

[6] Akishima Y, Ito K, Zhang L, Ishikawa Y, Orikasa H, Kiguchi H, Akasaka Y, Komiyama K, Ishii T (2004). Immunohistochemical detection of human small lymphatic vessels under normal and pathological conditions using the LYVE-1 antibody.. Virchows Arch.

[7] Cursiefen C, Chen L, Dana MR, Streilein JW (2003). Corneal lymphangiogenesis: evidence, mechanisms, and implications for corneal transplant immunology.. Cornea.

[8] Cursiefen C, Schlötzer-Schrehardt U, Küchle M, Sorokin L, Breiteneder-Geleff S, Alitalo K, Jackson D (2002). Lymphatic vessels in vascularized human corneas: immunohistochemical investigation using LYVE-1 and podoplanin.. Invest Ophthalmol Vis Sci.

[9] Collin HB (1974). A quantitative electron microscopic study of growing corneal lymphatic vessels.. Exp Eye Res.

[10] Ling S, Lin H, Xiang D, Feng G, Zhang X (2008). Clinical and experimental research of corneal lymphangiogenesis after keratoplasty.. Ophthalmologica.

[11] Cursiefen C, Cao J, Chen L, Liu Y, Maruyama K, Jackson D, Kruse FE, Wiegand SJ, Dana MR, Streilein JW (2004). Inhibition of hemangiogenesis and lymphangiogenesis after normal-risk corneal transplantation by neutralizing VEGF promotes graft survival.. Invest Ophthalmol Vis Sci.

[12] Dana MR (2006). Angiogenesis and lymphangiogenesis-implications for corneal immunity.. Semin Ophthalmol.

[13] Mouta C (2003). Inflammatory triggers of lymphangiogenesis.. Lymphat Res Biol.

[14] Banerji S, Ni J, Wang SX, Clasper S, Su J, Tammi R, Jones M, Jackson DG (1999). LYVE-1, a new homologue of the CD44 glycoprotein, is a lymph-specific receptor for hyaluronan.. J Cell Biol.

[15] Cursiefen C, Seitz B, Dana MR, Streilein JW (2003). Angiogenesis and lymphangiogenesis in the cornea: Pathogenesis, clinical implications and treatment options.. Ophthalmologe.

[16] Dana MRSJ (1996). Loss and restoration of immune privilege in eyes with corneal neovascularization.. Invest Ophthalmol Vis Sci.

[17] Cursiefen C, Maruyama K, Jackson DG, Streilein JW, Kruse FE (2006). Time course of angiogenesis and lymphangiogenesis after brief corneal inflammation.. Cornea.

[18] Chen L, Cursiefen C, Barabino S, Zhang Q, Dana MR (2005). Novel expression and characterization of lymphatic vessel endothelial hyaluronate receptor 1 (LYVE-1) by conjunctival cells.. Invest Ophthalmol Vis Sci.

[19] Yamagami S, Dana MR, Tsuru T (2002). Draining lymph nodes play an essential role in alloimmunity generated in response to high-risk corneal transplantation.. Cornea.

[20] Collin HB (1970). Lymphatic drainage of 131-I-albumin from the vascularized cornea.. Invest Ophthalmol.

[21] Smolin G (1971). Lymphatic drainage from vascularized rabbit cornea.. Am J Ophthalmol.

[22] Björndahl MA, Cao R, Burton JB, Brakenhielm E, Religa P, Galter D, Wu L, Cao Y (2005). Vascular endothelial growth factor-a promotes peritumoral lymphangiogenesis and lymphatic metastasis.. Cancer Res.

[23] Cao Y (2005). Direct role of PDGF-BB in lymphangiogenesis and lymphatic metastasis.. Cell Cycle.

[24] Ozerdem U (2006). Targeting of pericytes diminishes neovascularization and lymphangiogenesis in prostate cancer.. Prostate.

[25] Chang L, Kaipainen A, Folkman J (2002). Lymphangiogenesis new mechanisms.. Ann N Y Acad Sci.

